# Analysis of Microbial Diversity in South Shetland Islands and Antarctic Peninsula Soils Based on Illumina High-Throughput Sequencing and Cultivation-Dependent Techniques

**DOI:** 10.3390/microorganisms11102517

**Published:** 2023-10-09

**Authors:** Siqi Cui, Jie Du, Lin Zhu, Di Xin, Yuhua Xin, Jianli Zhang

**Affiliations:** 1 Key Laboratory of Molecular Medicine and Biotherapy, School of Life Science, Beijing Institute of Technology, Beijing 100081, China; candy9797979797@163.com (S.C.); dj535339752@outlook.com (J.D.); zhulin-2961@foxmail.com (L.Z.); cnd909@sina.com (D.X.); 2 China General Microbiological Culture Collection Center, Institute of Microbiology, Chinese Academy of Sciences, Beijing 100101, China; xinyh@im.ac.cn

**Keywords:** Antarctic soils, microbial diversity, 16S rRNA, Illumina sequencing, psychropoilic, novel taxa

## Abstract

To assess the diversity of bacterial taxa in Antarctic soils and obtain novel microbial resources, 15 samples from 3 sampling sites (DIS5, GWS7, FPS10) of South Shetland Islands and 2 sampling sites (APS18, CIS17) of Antarctic Peninsula were collected. High-throughput sequencing (HTS) of 16S rRNA genes within these samples was conducted on an Illumina Miseq platform. A total of 140,303 16S rRNA gene reads comprising 802 operational taxonomic units (OTUs) were obtained. After taxonomic classification, 25 phyla, 196 genera, and a high proportion of unidentified taxa were detected, among which seven phyla and 99 genera were firstly detected in Antarctica. The bacterial communities were dominated by *Actinomycetota* (40.40%), *Pseudomonadota* (17.14%), *Bacteroidota* (10.55%) and *Chloroflexota* (10.26%). Based on the HTS analyses, cultivation-dependent techniques were optimized to identify the cultivable members. A total of 30 different genera including 91 strains were obtained, the majority of which has previously been reported from Antarctica. However, for the genera *Microterricola*, *Dyadobacter*, *Filibacter*, *Duganella*, *Ensifer*, *Antarcticirhabdus* and *Microvirga*, this is the first report in Antarctica. In addition, seven strains represented novel taxa, two of which were psychropoilic and could be valuable resources for further research of cold-adaptability and their ecological significance in Antarctica.

## 1. Introduction

Most of Antarctica is covered by snow and ice; only about 0.4% of land area is permanently ice-free [1]. As a result of unique climatic and geographical characteristics like severe cold, strong wind and high radiation, microorganisms have an absolute advantage in the Antarctic soil ecosystem compared with plants and animals, playing a major role in the physical and chemical cycle. The traditional view is that extreme environments generally support biological communities with low biomass and low diversity [2]. However, after years of exploration and scientific research, it has been discovered that Antarctica is a huge treasure house of microbial resources, and the microbial diversity in its soil is much higher than previously thought [2,3,4,5]. Furthermore, Antarctic microorganisms have formed unique biomolecular structures and special physiological and biochemical properties in the long-term natural selection evolution. Many strains can produce low-temperature (cold-resistant) enzymes, as well as anti-radiation, anti-bacterial, anti-cancer active substances, which are very important in many fields including environmental engineering, agriculture, food industry, pharmaceutical industry, enzyme industry, biofuel, etc.

The South Shetland Islands are composed of a series of islands, the largest of which is King George Island, followed by Livingston and Deception Island. More than 80% of the archipelago was covered by sea ice from early April to early December. As glaciers are retreating in recent years, the regions closer to the sea are free of snow and ice, submitted to rapid cycles of freeze/thaw, and may receive significant quantities of organic material from marine animals [6,7]. There are many scientific research stations on the Fildes Peninsula of King George Island. A large number of nitrogen-fixing bacteria, nitrifying bacteria, and denitrifying bacteria have been isolated via traditional culture methods [8]. Then, more taxa in the soil near the Great Wall Station including *Pseudomonadota*, *Actinomycetota*, *Bacteroidota*, *Acidobacteriota*, *Cyanobacteriota*, and *Chloroflexota* were found via molecular biology methods. The Deception Island is formed by the crater collapse after the Antarctic submarine volcano erupted during the ancient glacial period [9]. Most ice-free areas on the island are covered by volcanic rocks and volcanic ash. The island also experienced volcanic eruption in the 1960s, leading to a stagnation of research on its microbial community in the next 30 years. The Antarctic Peninsula harbored a location further south (63° S) and a correspondingly colder climate. Affected by climate characteristics, geographical factors, and ecological environment, these five sampling sites may have unique microbial diversity and resources.

Up to now, only a small fraction of all Antarctic microbes has been isolated and characterized, which was limited by available technology and difficulty in sampling. 16S rRNA gene cloning and denaturing gradient gel electrophoresis (DGGE) have been traditionally used to identify uncultured microbial community structures in the past [10]. With the rapid technological innovation in molecular biology, High-throughput sequencing precludes the need to build clone libraries, and the efficiency associated with HTS sequencing is increasing and the widespread adoption of the Illumina Miseq sequencing platform has accelerated analytical capabilities [11,12].

Conducting microbial diversity analysis on soil in the Antarctic region not only reveals the structure of microbial communities in the region and the impact of environmental factors on microbial communities, but also facilitates the discovery of new species and enriches the gene pool of species. Moreover, digging and developing some strains with special active functions has important theoretical and practical significance, which has broad application prospects in the development of new bioactive substances and bioremediation due to the abundant microbial resources in Antarctica. In this context, the study aimed to (1) obtain a systematic understanding of the community structure and diversity of microorganisms inhabiting the soils from South Shetland Islands and Antarctic Peninsula; (2) optimize the isolation and cultivation strategies to explore the bacterial strains and to assess their bio-characteristics and potential functions in depth; and (3) explore novel taxa and enrich the gene pool to lay the foundation for the protection and utilization of Antarctic environmental microbial resources.

## 2. Materials and Methods

### 2.1. Sample Collection

**Figure 1:**
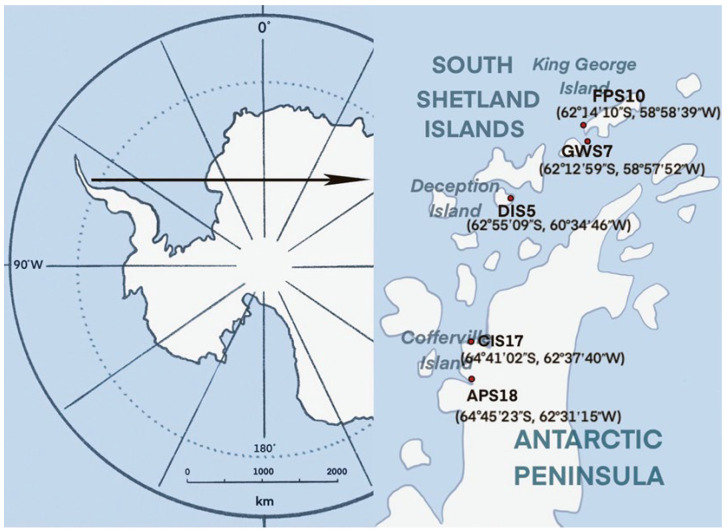
Location of South Shetland Islands and Antarctic Peninsula and the sampling sites. Three parallel settings for each sampling site, each 2 m apart.

Soil samples were collected from South Shetland Islands (Deception Island and King George Island) and Antarctic Peninsula in February, during the summer in Antarctica. The soil samples were collected at different latitudes and longitudes (Figure 1). According to the five-point sampling method, five sampling points were set up at different sites and three parallel samples were taken at each sampling point. Each sampling point was separated by 2 m. The surface debris was removed with a sterile spatula, and the samples were collected in sterile plastic tubes and shipped at −20 °C to laboratory. Once samples arrived at the laboratory, they were maintained at −80 °C until processing. Samples were selected from 5 sampling sites for this investigation. Samples from the same sampling site were mixed together for the experiment. The detailed information of the sampling sites is shown in Table 1 and the chemical and physical characteristics of the soil samples is shown in Table 2.

### 2.2. DNA Extraction, PCR Amplification and Sequencing

The soil DNA was extracted using the PowerSoil^®^ DNA Isolation Kit (MoBio, Solana Beach, CA, USA). The extracted genomic DNA was used as the template to amplify the V3–V4 region of 16S rRNA genes with primers 338 forward (5′-ACTCCTACGGGAGGCAGCA-3′) and 806 reverse (5′-GGACTACHVGGGTWTCTAAT-3′) [13]. The reaction components were 12.5 µL of 2×Taq PCR Mastermix (Promega; Madison, WI, USA), 1 µL of dNTP (10 mM), 3 µL of BSA (2 ng/μL), 1 µL of Forward Primer (5 μM), 1 µL of Reverse Primer (5 μM), X µL of DNA (30 ng), and 6.5-X µL of ddH_2_O. The PCR was carried out under the following thermocyling conditions: 95 °C for 5 min, followed by 25 cycles of 95 °C for 45 s, 50 °C for 50 s, 72 °C for 45 s, with a final extension at 72 °C for 5 min. This was repeated 3 times for each sample. The PCR products of the same sample were mixed at the same concentration and detected by 1% agarose gel electrophoresis. The AxyPrepDNA gel recovery kit (AXYGEN) was used to recover bands of 400–500 bp. The PCR products were detected and quantified through QuantiFluor™ -ST (Promega). The double-end sequencing analysis was carried out on the Illumina MiSeq PE300.

### 2.3. Sequence Processing and Analyses

Overlapping reads were merged using the program FLASH with default parameters [14]. QIIME [15] processing and the UCHIME arithmetic [16] were used and then effective tags were obtained. Operational taxonomic units (OTUs) were clustered by UPARSE [17] according to an open-reference OTU picking protocol based on 97% nucleotide similarity. The Venn diagram [18] made by the R platform was used to count the number of common and unique OTUs in multiple samples. Taxonomic relative abundance profiles (such as at the phylum, family and genus levels) were generated based on OTU annotation. Sequences obtained from this research were deposited in the NCBI SRA database (http://www.ncbi.nlm.nih.gov/traces/sra/, accessed on 25 July 2023): Bioprojects PRJNA681991.

### 2.4. Statistical Analysis

Statistical analysis was implemented using the R platform. Dilution curves and Shannon–Wiener curves were obtained through MOTHUR [19] and the R platform. Shannon index, phylogenetic diversity, Chao1 index, and the observed number of species were used to evaluate alpha diversity [20], and the weighted and unweighted UniFrac distances were used to evaluate beta diversity with QIIME (Version 1.7.0) [21,22,23]. Chao1 index and Shannon index were calculated at the lowest sequencing depth by random sampling using QIIME [24]. The relationships between bacterial community structures were evaluated by principal coordinate analysis (PCoA) based on the UniFrac distances between samples.

### 2.5. Isolation, Purification and Preservation of Strains

Bacterial strains were isolated by serial dilution and plating techniques to calculate the number of colonies in the sample and facilitate the cultivation and growth of bacterial colonies to obtain a single colony. Nine modified media (beef extract peptone, Gauze’s synthetic medium No.1, LB, R2A, TSBA, 1/10 TSBA, improved glycerol asparagin medium, Modified Gauze’s No.2, and MA) were adopted to optimize the cultivation of a broader range of soil microorganisms [25,26,27]. One gram of each sample was suspended in 9 mL of sterile water, homogenized in an incubator with shaking at 16 °C and 150 rpm for 1 h, and serially diluted from 10^−1^ to 10^−6^. Next, 0.1 mL of each dilution was spread onto nine media, respectively, and incubated at 4 °C and 28 °C for 15–20 days and 7–10 days, three plates at each temperature. After incubation, bacterial colonies were obtained from suitably diluted plates and transferred onto freshly prepared plates until pure cultures were obtained. The pure strains were maintained at 4 °C as slant and glycerol stock (20%) at −80 °C for further use.

### 2.6. Psychrophilic Bacteria Screening

The isolates were screened for temperature tolerance by incubating the culture spot inoculated plates at different temperatures [28]. The cultures obtained at 4 °C were screened further by inoculating at −4 °C, 0 °C, 4 °C, 10 °C, 15 °C, 20 °C, 25 °C and 28 °C for 3–14 days. All the tested strains were screened in triplicate and depending on the temperature range for optimal growth.

### 2.7. 16S rRNA Gene Sequencing and Data Analysis of Isolated Strains

DNA extraction was performed following the protocol described by Kim et al. [27] and Rainey et al. [29] with some modifications. The PCR reaction components were 25 µL of 2×PCR Mastermix (Promega; Madison, USA), 1 µL of dNTP (10 mM), 1 µL of Forward Primer (27F), 1 µL of Reverse Primer (1525R), 2 µL of DNA, and 20 µL of ddH_2_O. The PCR program consisted of an initial denaturation of 95 °C for 5 min, 34 cycles of 95 °C for 35 s, 54 °C for 60 s and 72 °C for 90 s, and a final extension step of 72 °C for 7 min. The PCR products were purified via the Wizard PCR Purification System (Promega), and the operation method was performed according to the procedure recommended in the instructions. Finally, sequencing was performed at Nuosai Genome Research Center (Beijing, China). The 16S rRNA gene sequences were compared with the EzBioCloud database [30] and GenBank databases using BLAST. The phylogenetic tree of 16S rRNA gene sequences of target strains and similar strains was constructed using the software of MEGA 6.0 [31].

## 3. Results

### 3.1. OTU Clustering and Annotation

**Figure 2:**
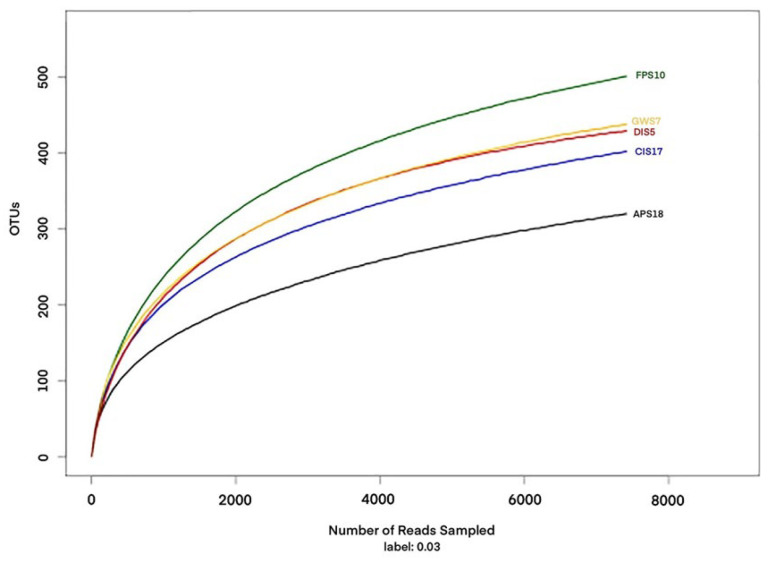
Rarefaction curves based on the sequences of the V3–V4 region of the 16S rRNA gene from all samples.

**Figure 3:**
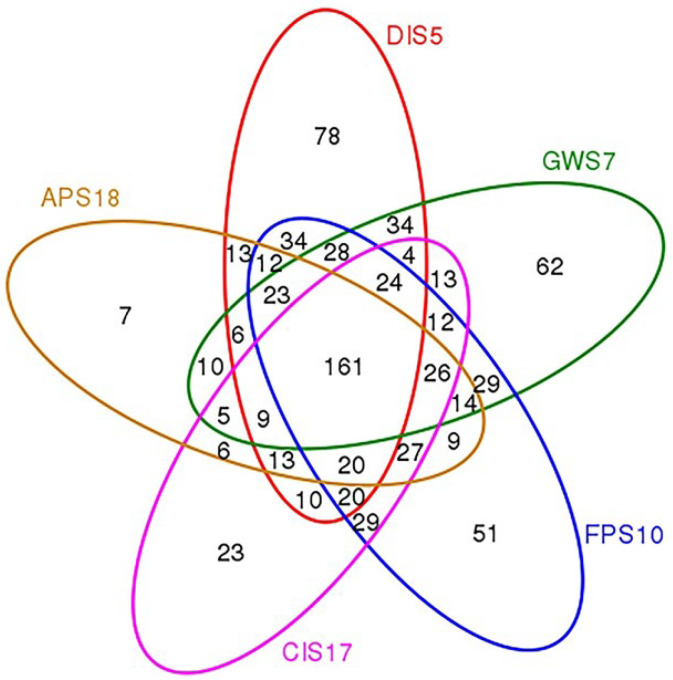
Venn diagram showing the OTUs shared among different samples.

A total of 140,303 sequences were obtained from the Illumina Miseq sequencing platform and 118,261 clean tags were determined to be of high quality, resulting in 802 OTUs (stringency at 97%). The number of high-quality reads per sample ranged from 16,539 to 27,577 (400–440 bp) (Table 3). The FPS10 sample harbored the highest number of OTUs, followed by DIS5, GWS7, CIS17 and APS18 (Table 3). Rarefaction curves (Figure 2), combined with the estimated coverage values (Table 3), suggested that the libraries were sufficiently large to capture a large majority of the bacterial diversity in the samples used in this study. A Venn diagram of the between-group OTUs was generated using the ggplot2 (Figure 3). Of 802 OTUs, 161 were common to all samples. The numbers of OTUs exclusive to the DIS5, GWS7, FPS10, CIS17 and APS18 samples were 78, 62, 51, 23, and 7, respectively.

**Figure 4:**
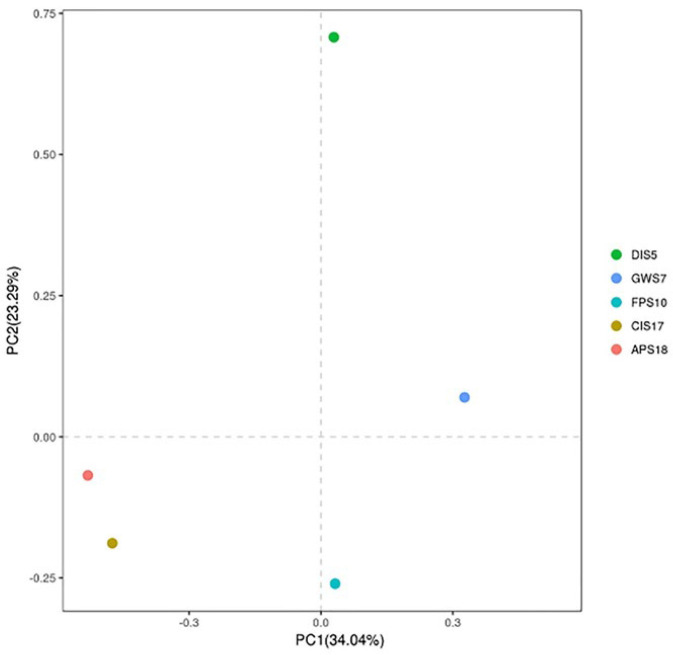
Hierarchical cluster analysis of communities in all samples.

The Chao1 index was used to estimate bacterial community richness and the Shannon–Wiener index was used to estimate bacterial community diversity in the five different samples (Table 3). The results indicated that both richness and diversity of the bacterial communities followed the same trend. The highest was for the King George Island, followed by Antarctic Peninsula and Deception Island. Samples were scattered among the three quadrants in the Principal Component Analysis (PCA) analysis plot. As shown in Figure 4, three samples in South Shetland Islands clustered together on the right of the coordinate axis, and two samples in the Antarctic Peninsula gathered together on the left of the coordinate axis. Communities in DIS5 were obviously deviated from those in the other four samples.

### 3.2. Composition and Relative Abundance of Microbiota

**Figure 5:**
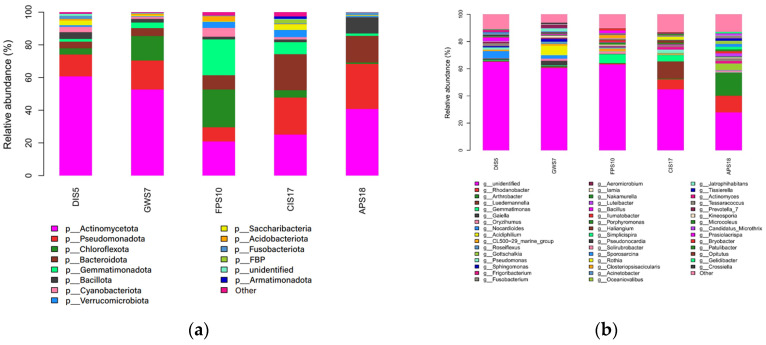
(**a**) Relative abundances of microbial communities at the phylum level in all samples. (**b**) Relative abundances of microbial communities at the genus level in all samples.

A total of 25 identified phyla and 196 identified genera were detected in samples. The phyla *Actinomycetota*, *Pseudomonadota*, *Chloroflexota*, *Bacteroidota*, *Bacillota*, *Nitrospirota*, *Cyanobacteriota*, *Saccharibacteria*, *Armatimonadota*, *Verrucomicrobiota*, *Gemmatimonadota* and *Acidobacteriota* were detected in all samples. However, their relative abundances varied across different samples. In the DIS5 sample, *Actinomycetota* (60.71%) and *Pseudomonadota* (13.37%) accounted for 74.08% of all bacteria. In the GWS7 sample, *Actinomycetota* (52.66%), *Pseudomonadota* (17.80%) and *Chloroflexota* (14.95%) represented 85.41% of all bacterial species. In the FPS10 sample, *Chloroflexota* (23.14%), *Gemmatimonadota* (21.93%) and *Actinomycetota* (20.92%) comprised 65.99% of the total microbiota. In the CIS17 sample, *Actinomycetota* (25.11%), *Pseudomonadota* (22.61%) and *Bacteroidota* (22.09%) accounted for 69.81% of all bacteria. In the APS18 sample, *Actinomycetota* (40.76%), *Pseudomonadota* (27.56%) and *Bacteroidota* (16.40%) comprised 84.72% of the total microbiota. The relative abundances of the top 14 phyla are shown in Figure 5a.

Overall, *Actinomycetota* was the predominant phylum in the DIS5, GWS7, CIS17 and APS18 samples, whereas in the FPS10 sample, *Chloroflexota* representatives were most abundant. In addition, the phylum *Chloroflexota* was more distributed on King George Island, and *Bacteroidota* accounted for high proportion in the Antarctic Peninsula samples.

The distribution of the microbiota at the genus level is illustrated in Figure 5b. In the DIS5 sample, the dominant genera included *Nocardioides*, *Bacillus*, *Fusobacterium* and *Pseudomonas*, which accounted for 5.21, 2.31, 1.90, and 1.66% of the microbiota, respectively. In the GWS7 sample, *Acidiphilium*, *Gaiella*, *Nocardioides* and *Prevotella* were the dominant genera, representing 6.89, 3.36, 2.55, and 2.03% of the microbiota, respectively. The FPS10 sample was dominated by *Gemmatimonas*, *Closteriopsisacicularis*, *Prasiolacrispa* and *Oryzihumus*, which contributed 6.45, 2.29, 1.86 and 1.66% to the total bacterial species, respectively. In the CIS17 sample, the dominant genera included *Luedemannella*, *Rhodanobacter*, *Gemmatimonas* and *Pseudomonas*, which accounted for 12.64, 7.27, 4.69 and 2.40% of the microbiota, respectively. In the APS18 sample, *Arthrobacter*, *Rhodanobacter*, *Gottschalkia* and *Sporosarcina* species represented 16.52, 12.15, 4.65 and 2.02% of the microbiota, respectively. These data indicated that in samples from South Shetland Islands, *Nocardioides*, *Acidiphilium* and *Gemmatimonas* were the most represented genera. *Rhodanobacter*, *Arthrobacter* and *Luedemannella* were the predominant genera in samples from Antarctic Peninsula. Additionally, unidentified genera represented 65.10, 60.59, 63.05, 44.82 and 27.89% of the microbiota in DIS5, GWS7, FPS10, CIS17 and APS18 samples, respectively.

It is noteworthy that seven phyla including *Gracilibacteria*, *Elusimicrobiota*, *Latescibacteria*, *Microgenomates*, *Parcubacteria*, *Saccharibacteria* and *Chlorobiota* and 99 genera (*Roseiflexus*, *Patulibacter*, *Perlucidibaca*, *Oceaniovalibus*, *Luteibacter*, *Rhizorhapis*, *Bryobacter*, *Frigoribacterium*, *Oryzihumus*, *Jatrophihabitans*, etc.) were firstly detected in Antarctica. In addition, many unclassified groups were detected in all samples, including even the group at the phylum taxon level. These results point out the high level of microbial diversity in Antarctica still to be explored.

### 3.3. Isolation of Cultivable Microorganisms

Ninety-one strains were isolated and purified from all soil samples, belonging to 4 phyla distributed in 30 genera (Table 4). Among them, *Actinomycetota* accounted for the highest proportion (34.07%), followed by *Bacillota* (31.87%), *Pseudomonadota* (30.77%) and *Bacteroidota* (3.29%), which presented results that were highly similar to those of the cultivation-independent analyses. At the genus level, *Bacillus* accounted for 21.98%, followed by *Pseudomonas* (10.99%), *Paeniglutamicibacter* (7.69%), *Sporosarcina* (6.59%), *Arthrobacter* (6.59%) and *Rhodococcus* (6.59%).

**Figure 6:**
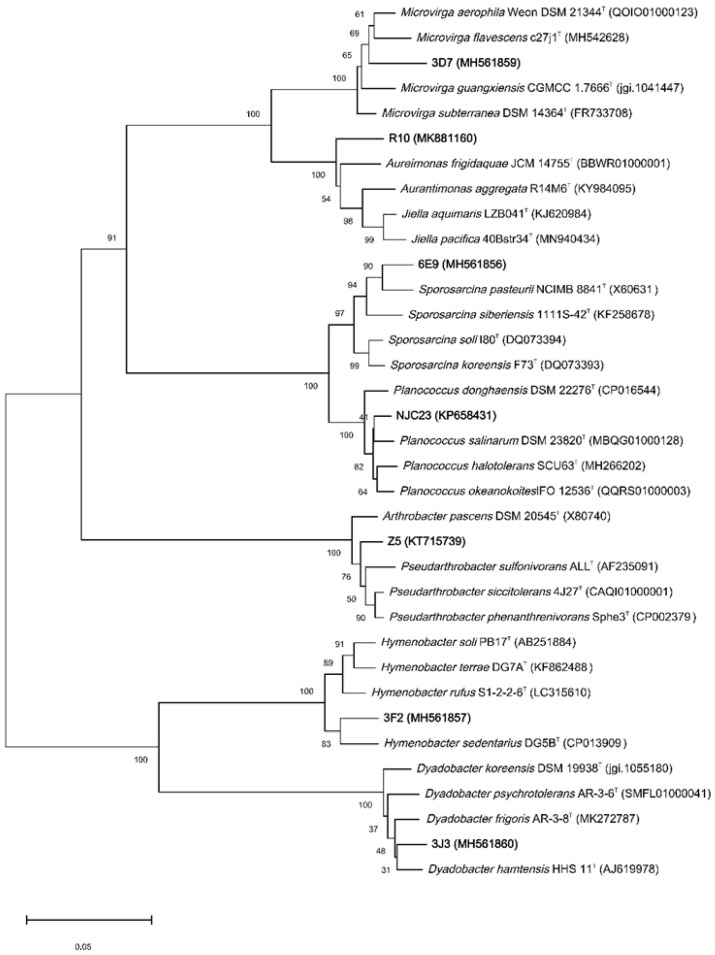
Dendrogram based on 16S rRNA gene sequences showing the relationship between seven novel isolates and their related type strains. Bar indicates 0.05 substitutions per nucleotide position.

A total of 41 strains in *Actinomycetota*, *Bacillota* and *Pseudomonadota* were obtained from DIS5, comprising 19 genera (Table 4). *Paeniglutamicibacter* and *Paracoccus* were the dominant genera, and they were not isolated from the other five samples. In comparison with previously reported results [2,3,4,5,32,33,34,35], four genera including *Microterricola*, *Dyadobacter*, *Microvirga*, *Ensifer* were firstly isolated from Antarctica. In addition, analysis based on 16S rRNA gene sequences revealed that strains 3F2, 3J3, 6E9, R10 and 3D7 shared less than 98.65% similarity to their most closely related species [36]. Then, we measured the full-length sequence of these strains and constructed phylogenetic trees, selected several standard strains for comparison based on the phylogenetic trees, measured various indicators such as physiological and biochemical characteristics, morphological characteristic, genetic analysis, and compared them with the standard strains. Based on phylogenetic, genotypic, chemotaxonomic, and phenotypic analyses, strains 3F2, 3J3, 6E9, and 3D7 represented novel species of the genus *Hymenobacter*, *Dyadobacter*, *Sporosarcina* and *Microvirga*, strain R10 represented a novel genus of the family *Aurantimonadaceae* (Figure 6).

Thirty strains were isolated from GWS7 and FPS10, comprising 4 phyla and 13 genera (Table 4). At the genus level, the dominant genera of GWS7 were *Pseudomonas*, *Rhodococcus* and *Sporosarcina*. The dominant bacteria in FPS10 were *Bacillus* and *Microbacterium*. The genus *Duganella* was isolated from Antarctica for the first time. Strain NJC23 represented a novel species of the genus *Planococcus* (Figure 6).

Twenty strains were isolated from the APS18 and CIS17, comprising three phyla and nine genera (Table 4). The dominant genera of CIS17 were *Bacillus* and *Pseudomonas*. Only five strains were isolated from APS18, among which the genus *Filibacter* was firstly obtained from the Antarctic. Strain Z5 represented a novel species of the genus *Pseudarthrobacter* (Figure 6).

### 3.4. Psychrophilic Members in Antarctic Soils

All 55 representative strains were obtained at 4 °C and screened further for tolerance to range of temperatures. Thirty-two strains were psychrophilic and exhibited an optimum temperature not above 20 °C. Most of the isolates recovered were pigmented and formed different -colored colonies (red, pink, orange, yellow, creamy-yellow and creamy white) on different media. Additionally, most strains grow well in relatively oligoltrophic media such as R2A and 1/10 strength TSB, indicating that there were many psychrophilic anatrophic-tolerant bacteria in Antarctic soils, which was consistent with our previous supposition. Importantly, among seven strains representing novel taxa, strains 3F2 and 3J3 could grow at −4 °C and exhibited the optimum growth temperature at 4 °C and 20 °C, respectively. These novel bacterial taxa are worthy of further bioprospecting studies.

## 4. Discussion

Over the last decade, rapid advances in molecular and cultural methodologies have started to realize some of the vast potential of Antarctic microbiology. The microbiota community of Grove Mountains soil Eastern Antarctica was analyzed previously by a traditional plate-culture technique, and 20 genera of *Bacillota*, *Pseudomonadota*, *Actinomycetota*, and *Bacteroidota* were isolated and identified. A large number of strains of *Acidobacteriota*, *Actinomycetota* and *Bacteroidota* were found in Dry Valleys soil of Antarctica, as well as a large proportion of unknown psychrophilic florac [2,32,35]. Despite continuing restrictions in spatial coverage, Antarctic microbiologists are now increasingly confident that Antarctic soil ecosystems harbor a rich bacterial community performing versatile ecological functions [32].

In this study, we performed Illumina high-throughput sequencing to determine microbiota in samples of South Shetland Islands and Antarctic Peninsula. A total of 25 phyla and 196 genera were detected though bioinformatics analysis. The major group identified were species of *Actinomycetota*, *Pseudomonadota*, *Chloroflexota*, and *Bacteroidota*, similar to previous studies in other geographic regions of Antarctica [2,4,5,35]. The widespread distribution of *Actinomycetes* in different Antarctic soils might be attribute to them being able to produce spores with high resistance to extreme environments such as dryness and extreme cold, and maintain a dynamic but dormant state in the form of spores for a long time. Unlike the previous studies, seven phyla and 99 genera including some rare genera like *Microterricola* and *Chryseolinea* were detected in Antarctica for the first time. Additionally, a fairly high proportion (27.89–65.10%) of unidentified genera were detected, supporting the idea that Antarctica is an excellent location to explore novel microorganisms, and very little is known about the huge treasure house [37].

Obvious differences in community structures were also observed among the samples. *Nocardioides*, *Acidiphilium* and *Gemmatimonas* represented the dominant genera in three samples from South Shetland islands, and *Luedemannella* and *Arthrobacter* dominated in two samples from Antarctic Peninsula. We found that the microbial diversity in the soils of King George Island and Deception Island was higher than the Antarctic Peninsula, which was consistent with the latter’s lower temperature due to its location further south. Recent studies demonstrated that the Antarctic soil microbial ecosystem is flexible and capable of rapid community adjustment in response to external environmental fluctuation [38,39]. The microbial groups in Deception Island soil samples were significantly different from other points, which may be related to its surface temperature, physical and chemical properties of the soil after the volcanic eruption and changes in the mineral elements. The Deception Island, as a special polar active volcanic island, was strongly influenced by the ocean, and had steep environmental gradients. Moreover, the soil of the Deception Island was a special case of extreme environments due to the geological factors and the volcanic eruption, where the microbial communities might have unique group compositions and survival strategies to resist nutritional deficiency and extreme temperatures. Its surface temperature, pH, salinity, and nutrient concentrations might together explain significant amounts of the variation in bacterial diversity. We speculated that the existence of these bacteria was related to the polar environment of the Deceptive Island and the geothermal activity during the volcanic period (volcanic activity, the marine environment, and the cryosphere). Furthermore, the Deception Island has high levels of heavy metals from its volcano, which might affect the diversity of microorganisms in this region. It was worth noting that most of the novel strains were isolated from here, indicating the huge potential for the development of microbial resources. Differences in microbial groups between soil samples at the Great Wall Station and the southern end of the Fildes Peninsula revealed the impact of human activities on microbial populations. Further studies will compare the diversity dynamics with seasonal changes as well as potentially identifying correlations with global warming.

Most microorganisms in nature are difficult to be cultivated in a laboratory. To tackle this problem, nine modified media were designed with the dilution plating method to isolate more strains in this study [40]. A total of 91 strains were obtained after culture separation, covering *Actinomycetota*, *Bacteroidota*, *Bacillota* and *Pseudomonadota*, which were highly similar to those of the cultivation-independent analyses. Among them, *Actinomycetota* mainly consisted of *Arthrobacter* and *Rhodococcus*, *Bacillota* mainly consisted of *Bacillus*, and *Pseudomonadota* mainly consisted of *Pseudomonas*. A considerable part of these genera were cold-resistant bacteria, including *Pseudomonas* sp., *Micrococcus* sp., *Flavobacterium* sp., *Bacillus* sp., etc. Seven new taxa belonging to *Pseudarthrobacter*, *Rhodoferax*, *Microvirga*, *Sporosarcina*, *Hymenobacter*, *Dyadobacter*, *Planococcus* and *Aurantimonadaceae* were obtained. Strain 3F2 grow at −4 °C–20 °C and exhibited the optimum growth temperature at 4 °C. Strain 3J3 grow at −4 °C–30 °C and optimally at 20 °C. They were highly resistant to ambient temperature and oligotrophic-tolerant.

In general, psychrophilic microorganisms exhibit higher growth yield and microbial activity at low temperatures compared to temperatures close to the maximum temperature of growth and has more often been put forth as an explanation to successful microbial adaptation to the natural cold environment [41]. Antarctica, represents cold deserts and a niche for cold-adapted microorganisms. Psychrophilic microorganisms have immense significance in the field of biotechnology because of their distinct metabolism from other organisms, which are also potential sources of novel pigments (as food additives), cold-active enzymes and antifreeze compounds.

Overall, this study provided valuable information regarding the diversity of bacteria inhabiting the Antarctic soils. Isolated strains of psychropoilic could not only constitute excellent models for the study of bacterial adaptation mechanisms to extremely cold conditions, but also could be used for future biotechnological applications.

## 5. Conclusions

In order to evaluate the diversity of bacterial communities in Antarctic soil and obtain new microbial resources, 15 samples were collected from three sampling sites (DIS5, GWS7, FPS10) in the South Shetland Islands and two sampling sites (APS18, CIS17) in the Antarctic Peninsula. High-throughput sequencing (HTS) of the 16S rRNA genes in these samples was performed on the Illumina Miseq platform. A total of 140,303 16S rRNA gene readings were obtained, including 802 operational taxonomic units (OTUs). Investigation showed that the dominant bacteria found in these samples belonged to *Actinomycetota*, *Pseudomonadota*, *Chloroflexota* and *Bacteroidota*. At the genus level, representatives of *Nocardioides*, *Acidiphilium*, *Gaiella*, *Gemmatimonas*, *Luedemannella* and *Arthrobacter* comprised most of the identified genera. Seven phyla and 99 genera were detected in Antarctica for the first time as well as a large number of unclassified groups. Ninety-one strains were isolated and identified from all samples using improved cultivation-dependent techniques. Most of them were cold-adapted and anatrophic-tolerant. Seven genera were isolated firstly from Antarctica. Six strains were identified as novel species of genera *Pseudarthrobacter*, *Hymenobacter*, *Dyadobacter*, *Planococcus*, *Sporosarcina* and *Microvirga*, and a strain was identified as novel genus of the family *Aurantimonadaceae*, two strains were psychrophilic. Most of the novel strains were isolated from the Deception Island, indicating the huge potential for the development of microbial resources. This study complemented the limited information available on the microbiology of Antarctic soils and helped attract attention on further development of Antarctic microbial resources. The isolated psychrophilic strains could also serve in future studies related to different fields of biotechnology.

## Figures and Tables

**Table 1:** The information of 5 samples collected from Antarctica.

Sample Name	Sample Color	Soil Sampling Location	Geographical Coordinates S/W
DIS5	black	Deception Island (pendulum bay)	62°55′09″/60°34′46″
GWS7	black brown	King George Island (Great Wall Station)	62°12′59″/58°57′52″
FPS10	yellowish-brown	King George Island (the southernmost tip of the Fildes Peninsula)	62°14′10″/58°58′39″
CIS17	black brown	Antarctic Peninsula (Cofferville island)	64°41′02″/62°37′40″
APS18	brown	Antarctic Peninsula	64°45′23″/62°31′15″

**Table 2:** The chemical and physical characteristics of the soil samples.

Sample Name	Carbon Contents	Nitrogen Contents	pH	Main Chemical Elements
DIS5	1.19%	0.16%	6.8	Fe, Mn
GWS7	0.73%	0.09%	6.3	Al, Ca, Mg, Fe
FPS10	0.70%	0.12%	6.1	Al, Cu, Fe
CIS17	10.96%	1.32%	5.8	Al, Fe, Cu, Zn
APS18	9.50%	1.33%	6.0	Al, Cu, Zn

**Table 3:** Numbers of clean tags, Operational taxonomic unit (OTU) richness and diversity indices of different soil samples with a 97% similarity cut-off.

Sample	Lean Tags	OTUs	Shannon	Chao1	Coverage (%)
DIS5	27,577	489	6.10	498	98.5
GWS7	23,542	460	6.97	545	98.1
FPS10	26,032	519	6.77	606	98.0
CIS17	24,571	361	5.96	427	98.5
APS18	16,539	402	6.52	506	98.3

**Table 4:** Bacteria recovered from different culture media from soil samples.

Phylum	Genus	Strain Number	Site	Closest Match	GenBank ID	Similarity (%)
*Actinobacteria*	*Arthrobacter*	LB8	DIS5	*Arthrobacter* *alpinus* DSM 22274^T^	GQ227413	98.98
		T10	DIS5	*Arthrobacter* *oryzae* NRRL B-24478^T^	CLG_48533	99.60
		D3	GWS7	*Arthrobacter* *pascens* DSM 20545^T^	X80740	99.58
		LB13, LB14, K11	DIS5	*Arthrobacter* *psychrochitiniphilus* GP3^T^	AJ810896	98.86
*Actinobacteria*	*Kocuria*	LB4	DIS5	*Kocuria* *palustris* DSM 11925^T^	Y16263	100.00
		C21	FPS10	*Kocuria* *rosea* DSM 20447^T^	X87756	99.73
	*Leifsonia*	T9	DIS5	*Leifsonia* *kafniensis* KFC-22^T^	AM889135	99.73
	*Microbacterium*	H3-2	FPS10	*Microbacterium* *aurum* KACC 15219^T^	CP018762	99.86
		O3	GWS7	*Microbacterium* *hydrocarbonoxydans* BNP48^T^	AJ698726	99.74
		C7, C8	FPS10, CIS17	*Microbacterium* *paraoxydans* NBRC 103076^T^	AJ491806	99.86
		B11	FPS10	*Microbacterium* *rhizomatis* DCY102^T^	KP161851	98.88
	*Micrococcus*	H3-1	FPS10	*Micrococcus* *aloeverae* AE-6^T^	KF524364	100.00
	*Microterricola*	LBN	DIS5	*Microterricola* *gilv* SSWW-21^T^	AM286414	99.04
	*Paeniglutamicibacter*	ON	DIS5	*Paeniglutamicibacter* *cryotolerans* LI3^T^	GQ406812	99.59
		T2, LB5	DIS5	*Paeniglutamicibacter* *sulfureus* DSM 20167^T^	X83409	99.58
		T3, T8, LB6	DIS5	*Paeniglutamicibacter* *sulfureus* DSM 20167^T^	X83409	99.45
		LB10	DIS5	*Paeniglutamicibacter* *sulfureus* DSM 20167^T^	X83409	98.62
	*Pseudarthrobacter*	Z5	APS18	*Pseudarthrobacter* *sulfonivorans* ALL^T^	AF235091	98.07
		H2	FPS10	*Pseudarthrobacter* *sulfonivorans* ALL^T^	AF235091	99.59
	*Rhodococcus*	L1	GWS7	*Rhodococcus* *fascians* LMG 3623^T^	X79186	100.00
*Actinobacteria*	*Rhodococcus*	H4	FPS10	*Rhodococcus* *kyotonensis* JCM 23211^T^	AB269261	99.18
		P1, B18, B21, E1	GWS7, CIS17, APS18	*Rhodococcus* *qingshengii* JCM 15477^T^	DQ090961	100.00
*Bacteroidetes*	*Flavobacterium*	NJA2	GWS7	*Flavobacterium* *circumlabens* CCM 8828^T^	AM177392	100.00
	*Hymenobacter*	3F2	DIS5	*Hymenobacter* *sedentarius* DG5B^T^	CP013909	97.03
	*Dyadobacter*	3J3	DIS5	*Dyadobacter* *koreensis* DSM19938^T^	jgi.1055180	98.08
*Firmicutes*	*Bacillus*	H1	FPS10	*Bacillus* *cereus* ATCC 14579^T^	AE016877	100.00
		T4, LB3, K18, O8	DIS5	*Bacillus* *oceanisediminis* H2^T^	GQ292772	99.30
		B16	CIS17	*Bacillus* *paralicheniformis* KJ-16^T^	LBMN01	100.00
		B14, C12, B22	FPS10, CIS17	*Bacillus* *paramycoides* NH24A2^T^	KJ812444	100.00
		C8	CIS17	*Bacillus* *siamensis* KCTC 13613^T^	GQ281299	99.46
		C4, B24	FPS10, CIS17	*Bacillus* *siamensis* KCTC 13613^T^	GQ281299	100.00
		C19	CIS17	*Bacillus* *siamensis* KCTC 13613^T^	GQ281299	99.86
		C22	FPS10	*Bacillus* *subtilis* subsp. *subtilis* NCIB 3610^T^	AJ276351	99.89
		B23, C14	CIS17, FPS10	*Bacillus* *wiedmannii* FSL W8-0169^T^	KU198626	100.00
		K20, C10, E2	DIS5, CIS17, APS18	*Bacillus* *zhangzhouensis* DW5-4^T^	JX680133	100.00
		C20	FPS10	*Bacillus* *zhangzhouensis* DW5-4^T^	JX680133	99.86
*Firmicutes*	*Filibacter*	W6	APS18	*Filibacter* *limicola* ATCC 43646^T^	AJ292316	98.77
	*Paenisporosarcina*	L10	DIS5	*Paenisporosarcina* *indica* PN2T^T^	FN397659	99.52
	*Planococcus*	NJC23	FPS10	*Planococcus* *salinarum* DSM 23820^T^	FJ765415	98.47
	*Sporosarcina*	K12,	DIS5	*Sporosarcina* *globispora* DSM 4^T^	X68415	100.00
		B30, B31, B32	GWS7	*Sporosarcina* *globispora* DSM 4^T^	X68415	99.86
		B7	APS18	*Sporosarcina* *globispora* DSM 4^T^	X68415	99.15
		6E9	DIS5	*Sporosarcina* *pasteurii* NCIMB 8841^T^	X60631	97.61
*Proteobacteria*	*Acinetobacter*	C24	FPS10	*Acinetobacter* *lwoffii* NCTC 5866^T^	X81665	99.86
	*Aurantimonas*	ON4, ON5	DIS5	*Aurantimonas* *endophytica* EGI 6500337^T^	KM114215	100.00
	*Duganella*	L8	GWS7	*Duganella* *zoogloeoides* IAM 12670^T^	D14256	98.65
	*Ensifer*	LB2	DIS5	*Ensifer* *meliloti* LMG 6133^T^	X67222	99.88
	*Janthinobacterium*	B28	CIS17	*Janthinobacterium* *lividum* DSM 1522^T^	Y08846	99.73
	*Jiella*	R10	DIS5	*Jiella* *aquimaris* LZB041^T^	KJ620984	96.35
	*Massilia*	C9	CIS17	*Massilia* *varians* CCUG 35299^T^	AM774587	99.34
	*Methylobacterium*	R12	DIS5	*Methylobacterium* *hispanicum* GP34^T^	AJ635304	99.86
	*Microvirga*	3D7	DIS5	*Microvirga* *subterranean* DSM 14364^T^	FR733708	96.75
*Proteobacteria*	*Paracoccus*	P13, R15, R17, LB12, LB1	DIS5	*Paracoccus* *aerius* 011410^T^	KX664462	100.00
		K15	DIS5	*Paracoccus* *marinus* KKL-A5^T^	AB185957	98.72
	*Pseudomonas*	P6, K3, C30	GWS7	*Pseudomonas* *caspiana* FBF102^T^	NR_152639	99.76
		P2	GWS7	*Pseudomonas* *frederiksbergensis* JAJ28^T^	AJ249382	99.17
		C15, B17	CIS17	*Pseudomonas* *gessardii* DSM 17152^T^	AF074384	100.00
		B13	CIS17	*Pseudomonas* *gessardii* DSM 17152^T^	AF074384	99.88
		R5	DIS5	*Pseudomonas* *mandelii* CIP 105273^T^	AF058286	99.17
		C29	GWS7	*Pseudomonas* *prosekii* LMG 26867^T^	LT629762	98.86
		C5	FPS10	*Pseudomonas* *prosekii* LMG 26867^T^	LT629762	99.43
	*Rhodoferax*	3D4	DIS5	*Rhodoferax* *koreense* DCY110^T^	CP019236	99.09
	*Sphingomonas*	G1	DIS5	*Sphingomonas* *panni* C52^T^	AJ575818	98.86

## Data Availability

The authors confirm that the data supporting the findings of this study are available within the article. The sequences generated during the current study are available in the NCBI SRA database (http://www.ncbi.nlm.nih.gov/traces/sra/, accessed on 25 July 2023): Bioprojects PRJNA681991. The 16S rRNA gene sequences of seven new strains (Z5, 3F2, 3J3, NJC23, 6E9, R10 and 3D7) were deposited in GenBank (https://www.ncbi.nlm.nih.gov/genbank/, accessed on 25 July 2023) under accession numbers KT715739, MH561857, MH561860, KP658431, MH561856, MK881160 and MH561859. Reference sequences used are noted in Table 4.
